# Urmia Lake (Northwest Iran): a brief review

**DOI:** 10.1186/1746-1448-3-5

**Published:** 2007-05-16

**Authors:** Amin Eimanifar, Feridon Mohebbi

**Affiliations:** 1 Iranian Artemia Research Center, P.O. Box: 57135-1367, Urmia, Iran; 2 Iranian Artemia Research Center, P.O. Box: 57135-368, Urmia, Iran

## Abstract

Lake Urmia (or Ormiyeh) is one of the largest hypersaline lakes in the world and the habitat of a unique bisexual *Artemia *species (*A. urmiana*). Despite this, and several other values of the lake, little literature on it has been published. The present paper is an attempt to provide a brief review on various aspects of the lake. Urmia Lake, located in northwestern Iran, is an oligotrophic lake of thalassohaline origin with a total surface area between 4750 and 6100 km^2 ^and a maximum depth of 16 m at an altitude of 1250 m. The lake is divided into north and south parts separated by a causeway in which a 1500-m gap provides little exchange of water between the two parts. Due to drought and increased demands for agricultural water in the lake's basin, the salinity of the lake has risen to more than 300 g/L during recent years, and large areas of the lake bed have been desiccated. Therefore, management and conservation of this incomparable ecosystem should be considered to improve the current condition by fisheries research institutes.

## Background

Lake Urmia (or Orumiyeh), is one of the largest permanent hypersaline lakes in the world and resembles the Great Salt Lake in the western USA in many respects of morphology, chemistry and sediments [1]. Despite this, and its several values, including conservation, little literature has been published on the lake and its biota

[2-7]. The aim of the present paper is to provide a brief review based on previous and recent literature on various aspects of Urmia Lake with particular emphasis on conservation and management. Although we cannot provide an all inclusive coverage here, we hope that it will be sufficient to introduce this remarkable lake to the general reader.

Urmia lake can be characterized as oligotrophic in terms of phytoplankton production in the range of 0.5–0.8 μg/l [6], with lower values compared to Great Salt Lake (0.5–3.5 μg/l) [8]. The predominance of the Na^+ ^and Cl^- ^ions illustrates the thalassohaline character of Urmia lake [9]. Therefore, Urmia Lake is an oligotrophic lake of thalassohaline origin [10] located in northwestern Iran at an altitude of 1250 m above sea level [6]. The total surface area ranges between 4750 km^2 ^and 6100 km^2 ^[11] depending on evaporation and water influx. The catchment area of the lake contains 21 permanent and ephemeral streams together with 39 episodic rivers, flowing through agricultural, urban and/or industrial areas that drain into this terminal lake, mostly without waste water treatment [12]. The maximum length and width of the lake are 128–140 km and 50 km, respectively [11,13]. The average and maximum depths are 6 m and 16 m, respectively [14]. The lake is divided into north and south parts separated by a causeway [15], which has a gap that allows for a limited exchange of water between the two arms [14].

Considering the role of *Artemia *in Urmia Lake, the Iranian Fisheries Research Organization (IFRO) established the *Artemia *Research Center in Urmia in 2000. On the basis of the vital role of *Artemia *as a live food in aquaculture, this center operates through various departments including Hydrochemistry, Stock Assessment, Culturing of Artemia and Algae species, Aquatic Diseases Hygiene. Researches about various aspects of *Artemia *in Urmia Lake are important research activities carried out at this center that has been nominated by FAO (Food Agriculture Organization) as a reference center for *Artemia *in central and west Asia. In addition, the *Artemia *& Aquatic Animals Research Center at Urmia University conducts various research projects on *Artemia *in collaboration with IFRO (Iranian Fisheries Research Organization). The IFRO *Artemia *Research Center seeks to achieve useful results in both basic and applied research on *Artemia *as a live-food organism for aquaculture.

## Geology

Most geological studies on Urmia Lake have been carried out on areas surrounding the lake [16], or on the geochemistry of the lake itself [17,18]. There are some geological formations which have been observed at different geographical areas of Urmia lake, namely; Jurassic limestone (northern areas), Eosin precipitation (southern areas), Igneous formations (eastern and northwestern areas), Metamorphic formations (northern, southwestern, southern, and southeastern areas), Oligocene formations (central areas), and Cretaceous formations (northern areas) [19]. Other aspects of the geology of the lake have been investigated by a few geologists [1,20]. The geology of the area consists of rocks from pre-cambrian to Quaternary, and very recent lake sediments [17]. Kelts and Shahrabi [1] suggested that palaeozoic metamorphic rocks constitute the western sides of the lake, while rocky cliffs of the eastern shores comprise Mesozoic flysch rocks. The northwestern shores, and many of the islands, are related to the lower Miocene which is represented by coralline limestone (Quom formation) [1].

## Sediments

The seasonal biologic cycle in Urmia Lake leads to the precipitation of aragonite in surface waters [1] induced by shifts in CO_2 _levels, due largely to biological activity [21]. Remarkably, the second most important component in the lake sediments consists of small rod-shaped fecal pellets of the brine shrimp *Artemia*. X-ray analyses show that the pellets are composed of 80 % aragonite along with small amounts of quartz and calcite, and rarely dolomite; true oolites have been found in only one locality in shoals along the western margin, north of Gulman Khaneh [1].

## Hydrology

The hydrology of wetland areas creates the unique physicochemical conditions that make such an ecosystem different from both well-drained terrestrial systems and deepwater aquatic systems. Hydrologic conditions are extremely important for the maintenance of a given water body's structure and function and affect many abiotic factors which, in turn, may impact the biota that develop in it [22]. Because saline lakes occur primarily in endorheic basins, they may be particularly sensitive to environmental changes because their size, salinity and annual mixing regimes vary with alterations in their hydrologic budgets [23,24].

## The basin

The total catchment area of the lake is about 51,876 Km^2 ^which is 3.15 % of that of the entire country, and includes 7 % of the total surface water in Iran [25]. There are thirteen main rivers in the lake basin, among them Zarrineh rood, the largest with a total annual discharge value of about 2×10^9 ^m^3 ^[12,17]. Climate in the Urmia Lake basin is harsh and continental, affected mainly by the mountains surrounding the lake [12,1]. Considerable seasonal fluctuations in air temperature occur in this semi arid climate with an annual average precipitation of between 200 and 300 mm [18,25].

The air temperature usually ranges between 0 and -20°C in winter, and up to 40°C in summer [26]. From this point of view, Urmia Lake is a critical asset for the region, because it acts to moderate these extremes [27]. Annual inflow into the lake is 6900×10^6 ^m^3^, of which 4900×10^6 ^m^3 ^is from rivers, 500×10^6 ^m^3 ^from flood water (through rainfall) and 1500×10^6 ^m^3 ^from precipitation [7,26]. Underground springs are also a source of water, but the volume is not known [5].

## Morphometry

**Figure 1:**
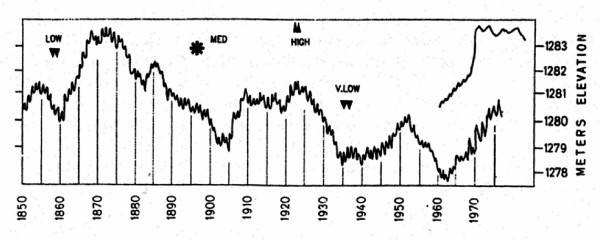
Water-level fluctuation curve for the Great Salt Lake (lower) compared with water-level information from Lake Urmia (upper data points) based partly on relative, historical observations. Note the sudden rise of 190 cm in Lake Urmia in spring 1969 resulting from the rapid surface runoff from exceptionally heavy winter snows.

Morphometric characteristics of Urmia Lake are among those features that have been reported variously by different authors [12,1,17]. In general, Urmia Lake has been shrinking for a long time, so its depth has decreased significantly during recent years. Due to 10 years of progressive dry climate in the area, the water level is 3 meters less than it was 20 years ago [28], a dramatic change. Little is known about historical lake-level variations because direct measurements have been sparse. Urmia lake had an increased level (2 m) in the winter 1968/1969 but the variations reached to an amount of 1284 m in 1977, with annual fluctuations of about 1 m (Fig. 1). There is a few data to show present lake-level variations, but the lake is recovering from a different water resources resulting from favorable climatic condition in 2007. Therefore, Urmia lake level reached to an amount of 1273 m and 25 cm, which was reported by West Azarbaijan (Urmia) Environment Organization.

However, highs and lows estimated from the odd casting-line measurement, or historical account [29-31] suggest an uncanny parallel with historical levels in the Great Salt Lake (Fig. 1) [1]. The water level of Great Salt Lake went down from 1999 to about 2003 due to less precipitation and increased from 2005 to the present because of a couple of higher precipitation years.

**Figure 2:**
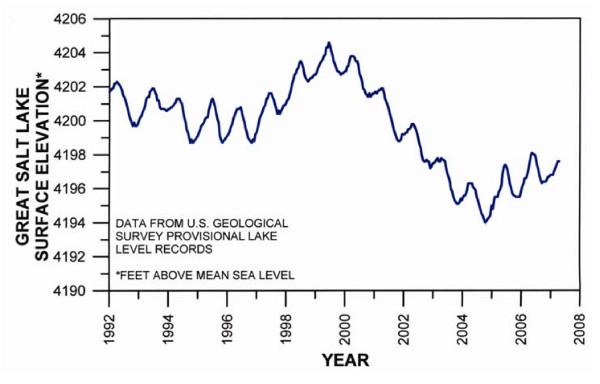
Annual variation of the surface elevation of south arm of Great Salt Lake, Utah (1992–2007). (Hydrograph from U.S. Geological Survey).

However, at present, the Great Salt Lake is not continuing its upward climb. The graph showed that Great Salt Lake elevation declined from 1992 to 1997 and then began an upward climb to 1999 (Fig. 2). The measurement of the level changes in Urmia lake by satellite showed an increase from 1992 to 1995 and decline from 1995 through 2003 (U.S. Geological Survey). A concomitant increase salinity (220–300 g/l) was observed during the years of declining elevation [32]. For the 2003–2004 period, favorable climatic changes lead to an increase in the elevation [32], but it has been declining since that time (U.S. Geological Survey). Aleshikh et al. [33] mapped the coastline changes for Urmia Lake using an innovative TM (Satellite imagery made up of 7 bands, with bands 1–5 and 7 being visible and near IR, and band 6 being thermal IR. The visible and near IR bands all have 30 m pixel size, with the thermal IR band having a pixel size of 120 m) and ETM+imagery (Satellite imagery is made up of 8 bands, with bands 1–5 and 7 being visible and near IR, band 6 being thermal IR, and band 8 being panchromatic. The visible and near IR bands all have 30 m pixel size, the thermal IR band has a pixel size of 60 m, and the panchromatic band has a pixel size of 15 m) method in 1989, 1998 and 2001. They concluded that the lake's small variations (0.2 m) from Jun-1989 to Aug-1998 are permanent, but that the great decrease in water depth from 1998 to 2001 (3 m), was not related to these permanent fluctuations, but to the lake's hydrologic budgets.

## Causeway project

A project to construct a highway across Urmia Lake was initiated in 1979 to facilitate communication between Tabriz in the east and Urmia in the west [15]. Martyr Kalantari highway divides the lake into south and north arms. To facilitate water flow between the north and south parts of the lake there is a 1400 m opening [6] covered by a bridge. Although most rivers flow into the lake from the south, and there is a continuous water flow from south to north, there is no evidence that the causeway has affected the circulating regime or salinity of the lake. In that connection it is relevant that [17] indicated that major ions were distributed homogenously throughout the lake due to the strong currents, despite the presence of the causeway across the lake. On the other hand, construction of the southern Pacific Railroad causeway partitioned the main body of Great Salt Lake into two bodies of dissimilar water, the north and south arms. The causeway caused three notable changes. First, the north arm has a much greater salinity than the south arm (currently about 60 percent higher). This is because the majority of the river inflow comes mainly into the south arm; the north arm receives less annual precipitation, and has a slightly higher evaporation rate than the south arm. The north arm receives mainly south-arm brine that flows south-to-north though a 300-foot-wide breach and two 15-foot wide culverts in the rock-fill causeway. There is minimal mixing between the two arms of the lake through the bi-directional flow that occurs through the same openings under the right hydrologic conditions. Second, the surface elevation of the south arm is higher than that of the north. The south arm is normally a 0.5 to 1-foot higher than the north arm, but was nearly 3.5 feet higher during the late 1980s flooding period. Third, the north arm currently supports a minimal brine shrimp density because of its extremely high salinity; where as the south arm has an abundance of brine shrimp. Before the construction of the causeway in 1959, brine shrimp thrived throughout the entire lake [34].

## Hydrochemistry

Generally, Urmia Lake is classified as oceanic, being of the sodium- chloride- sulfate type [35]. Most studies on chemical parameters of Urmia Lake are based on a few randomly selected samples; therefore, these results can not be used to provide an integrated scheme for seasonal and annual fluctuations of parameters in the lake.

## Main ions

The main cations in the lake water include Na^+^, K^+^, Ca^+2^, Li^+ ^and Mg^+2^, while Cl^-^, SO_4 _^-2^, HCO_3 _^- ^are the main anions [18]. The Na^+ ^and Cl^- ^concentration is roughly 4 times the concentration of natural seawater [9]. Sodium ions are at slightly higher concentration in the south compared to the north of the lake, which could result from the shallower depth in the south, and a higher net evaporation rate [36,37].

## Exploitation

Alipour [17] concluded that salts, except for halite, are generally lower in Urmia Lake compared to its sister, the Great Salt Lake in the USA, particularly those of K^+^. The latter consideration might make potassium recovery uneconomical in comparison to the Great Salt Lake. Sodium chloride has been obtained from solar evaporation ponds around Urmia Lake for a very long time, probably since the first humans populated the region. At present, 450 thousand tons of salt is recovered from the lake annually, of which four hundred thousand tons are used for production of NaCO_3 _in the city of Maragheh in the southeast corner of Urmia Lake, and the remaining fifty thousand tons are produced by small private operators for export and domestic use in villages around the lake [20,17]. In comparison, the salt industry at the Great Salt Lake generates more than 32,000 ha of solar-evaporation ponds for an annual production of more than two million tons of sodium chloride and other products (potassium sulfate, magnesium metal, chlorine gas, magnesium chloride products and certain nutritional supplements) all by a few large companies [38-41].

## Artemia biology

Hypersaline organisms adapt to high salinities by means of various physiologic mechanisms, including osmoregulation and the synthesis and accumulation of various compatible solutes. The brine shrimp, *Artemia *is the dominant macrozooplankton present in many hypersaline environments [42]. Their ability to survive and even thrive in forbidding environments has long been of interest to biologists [43,44]. This crustacean, properly called an animal extremophile, has been able to survive in such environments through well-developed osmoregulation involving enhanced Na-K ATPase activity [45]. Although physiologically able to survive and reproduce in salinities near and below seawater, *Artemia *is definitely found at salinities below 100 gl^-1 ^but the density maybe decreased [46,47].

## Bisexual vs. parthenogenetic Artemia

The first description of *Artemia *was carried out in Lymington (England) by Schlosser in 1755 [48]. Since then, *Artemia *has been reported from very many sites around the world. The latest study on *Artemia *biogeography has been published by [49], who reported 598 sites, spread over the five continents. Although, the presence of *Artemia *in Urmia Lake was first reported over a century ago [29], a long period elapsed before this population was characterized [14]. Günther [50] described brine shrimp from Urmia Lake as a unique bisexual species, a characteristic confirmed by [51]. These researchers demonstrated the reproductive isolation of the species from other bisexual strains and assigned the binomen *Artemia urmiana*. However, [52] and [53], based on cytogenetics and repetitive DNA and heterochromatin analysis, reported that the Urmia Lake population was exclusively parthenogenetic. As a result, Barigozzi and Baratelli [54] proposed to cancel the binomen *Artemia urmiana*. At the same time, Takami [55] reported the co-occurrence of both bisexual and parthenogenetic *Artemia *in Urmia Lake. Subsequent studies by Browne and Bowen [56] suggested that both sexual and asexual populations exist in Urmia Lake. According to the most recent studies [14] parthenogenetic populations are found at restricted coastal areas of Urmia Lake, which supports the earlier findings of Agh [57] and suggestions made by Sorgeloos [58].

A variety of hypersaline environments exist in Iran with the potential existence of *Artemia *in those regions. However, little research has been carried out on the characteristics of these *Artemia *populations. Various authors [59-61] have reported the occurrence of *Artemia *in other regions of Iran. Most recently, Abazopoulos et al. [14] investigated *Artemia *sites and populations around Iran and showed that *Artemia *is present at 17 different locations, all of these populations being parthenognetic, except for *Artemia urmiana *in Lake Urmia. Biometrical and biochemical analyses have been performed on cysts and stage-I nauplii hatched from cysts collected from seven sites in Urmia Lake by Abazopoulos et al. [62]. They carried out buoyancy tests and performed transmission electron microscopy on both *Artemia urmiana *(Iran) and *Artemia franciscana *(USA) cysts.

## Ecology

Compared to other aspects of brine shrimp biology, ecological studies on *Artemia *are relatively few in number [46], and that is especially the case for Urmia Lake. The brine shrimp population in Urmia Lake exhibits seasonal fluctuations that are similar to those reported for the Great Salt Lake [8] but differ in significant ways. Thus, hatching of cysts in the Great Salt Lake may occur as early as February when grazing pressure prevents the phytoplankton from reaching actual blooming densities in late Spring as observed in other saline habitats [6]. Adult *Artemia *densities in Urmia Lake (3 adult. L^-1^) [6] in July 1994-January 1996 can be compared to the values reported for Great Salt Lake (4 adults. L^-1 ^in July) [63] and 20 and 10 L^-1 ^for June and July, respectively [64] and 10–20 L^-1 ^in late spring [65], respectively. These low densities correlate well with low algal biomass, and agree with the findings of [66] that in the Great Salt Lake the highest *Artemia *densities coincide with the highest food concentrations, clearly illustrating the impact of food availability on the growth and reproduction of *Artemia*.

## Phytoplankton

Some contradictions have appeared in the literature concerning the phytoplankton populations of Urmia Lake. For example, *Enteromorpha intestinalis*, a macroscopic green alga, was reported by [67] to occur at one sampling site. In years when salt concentrations remain low, Enteromorpha becomes so abundant that the whole lake takes on the appearance of a thin vegetable soup.

On the other hand, various authors have reported relatively different phytoplankton populations from different sampling sites of the lake. For example, Ryahi et al. [68] observed 6 cyanophyta (*Anabaena *sp.; *Anacystis *sp.; *Chrococcus *sp.; *Lyngbya *sp.; *Oscillatoria *sp. and *Synechocuus *sp.), 4 Chlorophyta (*Ankistrodesmus *sp.; *Dunaliella *sp.; *Monostroma *sp. and *Pandorina *sp.), 2 bascillariophyta (*Amphora *sp. and *Navicula *sp.). Mohebbi et al. [69] reported 3 cyanophyta (*Anabaena *sp.; *Oscillatoria *sp. and *Synechoccus *sp.), 2 Chlorophyta (*Dunaliella *sp. and *Ankistrodesmus *sp.), 11 bascillariophyta (*Navicula *sp.; *Nitzschia *sp.; *Cyclotella *sp.; *Symbella *sp.; *Synedra *sp.; *Pinnullaria *sp.; *Diatoma *sp.; *Amphiprora *sp.; *Surirrela *sp.; *Cymatopleura *sp. and *Gyrosigma *sp.), during monthly samplings over an entire year. These variations may be related to limited and irregular sampling, or increased salinity of the lake during recent years that has eliminated some non- tolerant species. Further work is clearly needed to resolve these matters.

Quantitative analysis of chlorophylls, and algal density, indicated that primary production in Urmia Lake is lower than that of its sister Great Salt Lake [8] and that *Dunaliella *is the dominant phytoplankton (more than 95 % of the total phytoplankton in number) of Urmia Lake [69,6]. Microalgae species composition of Urmia lake is roughly similar to the phytoplankton in Great Salt lake, that consists predominantly of *Dunaliella*, with an important fraction of diatoms like *Navicula *and *Nitzschia *[9].

## Bacteriology

Ghaheri and Baghal-Vayjooee [12] in their summary review of Urmia Lake referred to bacteriological tests conducted on lake sediments and concluded that there are no bacteria in the lake that are pathogenic to humans. These authors did mention the presence of non-pathogenic bacteria, detected using dead *Artemia *remains. In previous studies performed by [67] two pathogenic bacteria, *Clostridium perfringens *and *Streptococcus faecalis *were detected in Urmia Lake. It seems that these bacteria exist mainly in those areas at which rivers enter into the lake and appear to have origins from agricultural runoff and possibly sewage systems.

## Conservation and management

Only relatively recently have the true value of wetlands been recognized [22]. There are a few wetlands which are located around the south part of Urmia lake namely, Kani Berazan, Hasanloo, Soldoz, Dorgeh Sangi, Goppi Baba Ali. In general, wetlands functions such as flood and storms control are the result of the interaction between their physical, chemical and biological compartments. The wetlands have considerable roles in production of natural resources which in turn will develop agricultural and industrial activities. The wetlands considered as suitable place for living aquatic organisms, and avian faunae therefore, accounted as incomparable ecosystems. Social and cultural importance in absorbing tourists will be other impact of wetlands [70]. The marshes have typical saltmarsh plant communities with Juncus, some Phragmites reed-beds at river mouths, and some Tamarix stands. During past decades, the salinity of many large and permanent salt lakes has risen due to human activities [71,72], and several have dried completely. These examples include Owens Lake in California [73], and Winnemucca Lake in Nevada [74]. In almost all cases, the reason for this desiccation is the diversion of inflowing rivers to meet agricultural and other human needs.

Urmia Lake with its endemic *Artemia *species and its large area (one of the largest natural habitats of *Artemia *in the world) has been proposed by UNESCO to become a national park [25] owing to its unique features and the recognition that it is an important natural asset, with considerable cultural, economic, aesthetic, recreational, scientific, conservation and ecological value. The lake is extremely important for breeding *Pelecanus onocrotalus*, *Egretta garzetta*, *Plegadis falcinellus*, *Platalea leucorodia*, *Phoenicopterus ruber*, *Tadorna ferruginea*, *Tadorna tadorna*, *Himantopus himantopus*, *Recurvirostra avosetta*, *Tringa totanus*, *Larus cachinnans armenicus *and *Larus genei*. Other breeding birds include several pairs of *Anser anser*, *Marmaronetta angustirostris *and *Aythya nyroca*. *Charadrius leschenaultii *has been recorded during the summer months and may breed on the saline flats around the lake. Flamingos are known to breed in large numbers at lake Orumiyeh, and numbers still appear to be increasing slightly, with perhaps as many as 25,000 breeding pairs in recent years. Towards the end of the breeding season, the adults congregate in huge rafts to moult. The vast mudflats surrounding the lake are the most important autumn staging area for migratory shorebirds and garganey Anas querquedula in Iran. The lake appears to be an important moulting area for common shelduck Tadorna tadorna.

The lake is marked by more than one hundred small rocky islands, which are stopover points in the migration of various waterfowl (including flamingos, pelicans, spoonbills, ibises, storks, shelducks, avocets, stilts and gulls) [75]. Stands of pistachio *Pistacia atlantica *woodland survive on the larger islands, notably on Ashk and Kabudan. Other conspicuous plants on those islands are buckthorn *Rhamnus *sp., species of wormwood Artemisia, Dianthus and grasses Hordeum and Bromus. The islands in Lake Urmia are the only known breeding site for lanner *Falco biarmicus *in Iran (at least five pairs), and also provide nesting sites for at least ten pairs of *Neophron percnopterus*. *Falco cherrug*, *Gyps fulvus*, *Aegypius monachus *and *Falco peregrinus *have been recorded during the summer months as visitors from the surrounding hills; and *Haliaeetus albicilla *and *Falco columbarius *occur in winter. A population of about 25 great bustards Otis tarda frequents the area, and breeds there (at least 4 pairs). Wild sheep *Ovis ammon *were introduced on Kabudan Island in the 19^th ^century, while Mesopotamian fallow deer Dama dama mesopotamica were introduced on Ashk in the 1970s.

But the salinity of Urmia Lake has increased dramatically to more than 300 g/l during recent years, and that has greatly influenced almost all aspects of the lake.

Population genetic studies which carried out by Eimanifar [3] showed that the south area caused most differentiated populations and the south and median areas of lake have relatively high genotypes (composite haplotypes) distribution as compared to north area.

The possible causes of rising salinity are likely to be surface flow diversions, groundwater extractions and unsuitable climate condition. About 4.4 million people live in the Urmia Lake basin, whose irrigation economy is strongly dependent on existing surface and groundwater resources in the area [25]. Accordingly, human population growth in the lake's basin has seriously increased the need for agricultural and potable water in recent years, all of which are supplied from surface and groundwater sources in the area.

These matters, together with poor weather conditions, have reduced significantly the volume of water entering the lake so that, at present, Urmia Lake has shrunk significantly and large areas of the former lake bed have been exposed. Diverting potential rivers such as Zab with high inflowing capability (mean water discharge = 44.52 m^3^/sec) [19] maybe useful for recovery of Urmia lake. Without such intervention, significant alterations in the biota of the lake will likely continue, and agricultural production may be dramatically and negatively impacted, including the unique Urmia Lake *Artemia*. It is important that conditions be maintained that enable this species to complete its reproductive cycle and thereby conserve this genetic resource. Attention must be given to the nature of threats and impact of human activities. An awareness and better understanding of the lake by the surrounding human population will provide a foundation for future sustainable management of this remarkable inland lake.
